# 

**Published:** 1893-12

**Authors:** 


### CONTENTS—DECEMBER, 1893.—VOL. IX., NO. 6.

ORIGINAL COMMUNICATIONS—
Castration of Sexual Perverts. F. E. Daniel, M. D . . . . . . . . . 255
Discussion on Same . . . . . . . . . . . . . . . . . . . . . . . . . 268
Otitis Media; Its Causes and Treatment. Robt. E. Moss, M. D., San Antonio . . . . . . . . . . . . . . . . . . . . . . . . . . 271
ABSTRACTS OF CURRENT MEDICAL LITERATURE—
PRACTICE OF MEDICINE—Treatment of Carbuncle by Injections of Carbolic Acid, 276; Creasote in the Treatment of Phthisis Pulmonalis, 277; Diagnosis of Typhoid Fever, 278; Yellow Fever from a Medico-Geographical and Prophylactic Point of View, 279.
THERAPEUTICS.—Ammonium Chloride in Cystitis, 280; Chloroform as a Tænicide, 281; Death from the Use of Pental, 281; Salicylic Acid in Rheumatism, 282; The Treatment of Chlorosis, 282; Large Doses of Strychnine in Pulmonary and Cardiac Diseases, 283.
GYNECOLOGY.—Papillary Cyst of the Paro-ophoron, 284; Fifty-nine Consecutive Operations for Suppurating Peri-Uterine Lesions, 285; Tardy Labor Due to Softness of the Fœtal Cranium, 286; Pelvic Abscess, 287; Hysterectomy vs. High Amputation of Cervix for Cancer, 288; Etiology of Ectopic Gestation, 289.
SOCIETY NOTES—
Southeast Texas Medical Society, 289; Austin District Medical Society, 290; Galveston County Medical Society, 290.
EDITORIAL—
Christmas Greeting . . . . . . . . . . . . . . . . . . . . . . . . . 291
Insurance and Assurance . . . . . . . . . . . . . . . . . . . . . . 292
Straining at a Gnat . . . . . . . . . . . . . . . . . . . . . . . . 297
Diagnosis of Diphtheria . . . . . . . . . . . . . . . . . . . . . . 298
Not a Stockholder . . . . . . . . . . . . . . . . . . . . . . . . . 299
MEDICAL NEWS AND MISCELLANY—
A Whole Lot of Interesting Paragraphs . . . . . . . . . . . . . . . 299
Evils of Substitution . . . . . . . . . . . . . . . . . . . . . . . 303
BOOK NOTICES . . . . . . . . . . . . . . . . . . . . . . . . . . . 307
PUBLISHERS' NOTES . . . . . . . . . . . . . . . . . . . . . . . . . 308

